# Revealing the missing expressed genes beyond the human reference genome by RNA-Seq

**DOI:** 10.1186/1471-2164-12-590

**Published:** 2011-12-02

**Authors:** Geng Chen, Ruiyuan Li, Leming Shi, Junyi Qi, Pengzhan Hu, Jian Luo, Mingyao Liu, Tieliu Shi

**Affiliations:** 1 Center for Bioinformatics and Computational Biology, and the Institute of Biomedical Sciences, School of Life Science, East China Normal University, Shanghai 200241, China; 2 National Key Laboratory of Crop Genetic Improvement, Huazhong Agricultural University, Wuhan, 430070, China; 3 National Center for Toxicological Research, US Food and Drug Administration, Jefferson, Arkansas, 72079, USA; 4 Shanghai Information Center for Life Sciences, Shanghai Institutes for Biological Sciences, Chinese Academy of Science, Shanghai 200031, China

## Abstract

**Background:**

The complete and accurate human reference genome is important for functional genomics researches. Therefore, the incomplete reference genome and individual specific sequences have significant effects on various studies.

**Results:**

we used two RNA-Seq datasets from human brain tissues and 10 mixed cell lines to investigate the completeness of human reference genome. First, we demonstrated that in previously identified ~5 Mb Asian and ~5 Mb African novel sequences that are absent from the human reference genome of NCBI build 36, ~211 kb and ~201 kb of them could be transcribed, respectively. Our results suggest that many of those transcribed regions are not specific to Asian and African, but also present in Caucasian. Then, we found that the expressions of 104 RefSeq genes that are unalignable to NCBI build 37 in brain and cell lines are higher than 0.1 RPKM. 55 of them are conserved across human, chimpanzee and macaque, suggesting that there are still a significant number of functional human genes absent from the human reference genome. Moreover, we identified hundreds of novel transcript contigs that cannot be aligned to NCBI build 37, RefSeq genes and EST sequences. Some of those novel transcript contigs are also conserved among human, chimpanzee and macaque. By positioning those contigs onto the human genome, we identified several large deletions in the reference genome. Several conserved novel transcript contigs were further validated by RT-PCR.

**Conclusion:**

Our findings demonstrate that a significant number of genes are still absent from the incomplete human reference genome, highlighting the importance of further refining the human reference genome and curating those missing genes. Our study also shows the importance of *de novo *transcriptome assembly. The comparative approach between reference genome and other related human genomes based on the transcriptome provides an alternative way to refine the human reference genome.

## Background

The latest version of the public human genome assembly NCBI build 37 (also known as GRCh37) has been released and is considered to be the successor to NCBI Build 36. Currently, different types of human genetic variation studies including single-nucleotide polymorphisms (SNPs), deletions, insertions and structure variation are all based on the human reference genome [1-6]. Moreover, the reference genome is the fundamental resource for the researches in genomics and transcriptomics, such as investigating the transcriptional structure of genes, inferring splicing patterns and quantifying the expression level of each transcript [7-10]. Therefore, the completeness of human reference genome is crucial for comprehensively understanding the structure of human genome.

Although the annotation information on the human reference genome has provided important information in the study of evolutionary history, population diversity, gene regulatory mechanisms and various biological events, it is still incomplete. In previous studies, Khaja et al. [11] and Kidd et al. [12] have reported that a notable portion of human genomic sequences were absent from NCBI build 35 or build 36, suggesting that the updated human reference genome is still not completely assembled and annotated. During the processes of mapping transcriptome sequencing reads onto the human reference genome to quantify the gene expression and infer alternative splicing or transcripts, the reads that cannot be mapped onto the human reference genome are usually simply discarded. In fact, some of those unmapped reads could be generated from certain functional genes that not present in the reference genome. Simply discarding such sequences can result in the loss of important information.

By comparing to the human reference genome NCBI build 36, Li et al. also identified a significant amount of Asian (YH) and African (NA18507) novel sequences that not present in NCBI build 36, and they speculated that most of those novel sequences are specific to Asian and African [13]. The new version of human reference genome NCBI build 37 resolves some problems existed in NCBI build 36 and provides us the opportunity to further explore these potential Asian (YH) and African (NA18507) novel sequences. Besides, the high sequencing throughput and feasibility of the sequencing technology for transcriptome [14-17] allow us to better understand the genome structure of the human genome. Here we further investigated the completeness of human reference genome and revealed a significant number of human genes and novel transcript contigs that are absent from NCBI build 37.

Our findings show that the Asian (YH) and African (NA18507) novel sequences that are absent from NCBI build 36 contain a significant portion of regions that can be transcribed. And our results indicate that many of those transcribed sequences are not specific to Asian (YH) and African (NA18507), but are also present in Caucasian populations. We also found that about half of 176 RefSeq genes that are unalignable to the human reference genome are expressed in human brain or cell lines. In addition, we identified hundreds of novel transcript contigs in human brain and cell lines through *de novo *assemblies of brain and cell line transcriptome. Our study suggests that the human reference genome is still missing a significant number of genes that can be transcribed and many of those missing genes are likely functional. With the help of *de novo *transcriptome assembly, we can further detect the missing information of the reference genome and better understand the structure of human genome. The comparative approach of genome sequences between reference genome and other related human genomes based on the transcriptome provides an alternative approach to refine the human reference genome.

## Results

### Detecting transcribed regions in Asian and African novel sequences

**Figure 1:**
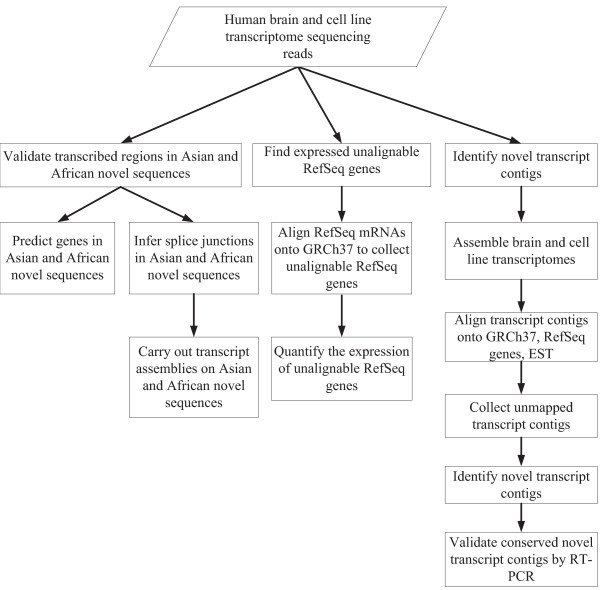
**Overview of identification of the missing expressed genes beyond the human reference genome**. Human brain and cell transcriptome sequencing reads were used to validate the transcribed regions in Asian and African novel sequences, quantify the expression of unalignable RefSeq genes and identify novel transcript contigs.

We used two transcriptome sequencing datasets from two reference RNA samples established by the MicroArray Quality Control (MAQC) project [18] with Illumina next-generation sequencing technology to carry out our study (Figure 1). The two reference RNA samples consist of the Universal Human Reference RNA (UHRR or UHR) from 10 human cell lines of various origins [19] and the Human Brain Reference RNA (HBRR or HBR) from several regions of the brain of 23 adult donors (22 of these donors are Caucasian, one is unknown). The two datasets consist of ~70.1 million and ~58.6 million of sequencing reads, respectively, with read-lengths of 100 bp (see Methods).

In a previous study, by comparing to the human reference genome NCBI build 36 with > 100 bp long and < 90% identity criteria, Li et al. identified ~5 Mb of Asian (YH) and ~5 Mb African (NA18507) novel sequences were not present in NCBI build 36, and they inferred that most of those novel sequences are individual or population specific [13]. To examine how many of these Asian (YH) and African (NA18507) novel sequences have been incorporated into GRCh37, we aligned [20] these novel sequences to GRCh37. Using 90% identity and 98% coverage as threshold, we found that only 991.5 kb (19.35%) Asian (YH) and 926.7 kb (19.31%) African (NA18507) novel sequences could be aligned to GRCh37, and the rest were still unalignable to GRCh37. The results suggest that those alignable Asian (YH) and African (NA18507) novel sequences are part of gaps or unsolved issues in NCBI build 36.

To verify whether genes contained in Asian (YH) and African (NA18507) novel sequences are expressed in brain and/or cell lines, we first predicted the genes in these novel sequences using Augustus [21]. Predictive results showed that 260 and 140 genes (including the incomplete genes at the sequence boundaries) are contained in Asian (YH) and African (NA18507) novel sequences, respectively. To further verify the prediction, we mapped [22] brain and cell line 100 bp reads onto the Asian (YH) and African (NA18507) novel sequences respectively to identify the splice junctions between the exons [23]. We inferred that there were 542 and 1,005 junctions in Asian (YH) novel sequences, whereas 133 and 236 junctions existed in African (NA18507) novel sequences, respectively.

We next carried out transcript assembly and quantification [24] using Asian (YH) and African (NA18507) novel sequences as reference sequences. We obtained 1,027 brain (100 bp ~ 2,335 bp in length) and 1,146 cell line (100 bp ~ 1,146 bp) transcripts from Asian (YH) novel sequences (Additional files 1 and 2). Meanwhile, we identified 887 brain (100 bp ~ 6,926 bp) and 947 cell line (100 bp ~ 1,897 bp) transcripts from African (NA18507) novel sequences (Additional files 3 and 4). Those transcripts were the assembled fragments generated from Cufflinks [24]. In the Asian (YH) novel sequences, the brain transcripts covered 40 predicted genes and the cell line transcripts covered 44 predicted genes, with 27 predicted genes in common to both samples. In the African (NA18507) novel sequences, brain transcripts covered 23 predicted genes and cell line transcripts covered 26 predicted genes, of which 18 predicted genes were in common. We also found that 246 brain and 249 cell line transcripts from Asian (YH) novel sequences could be aligned to GRCh37; and 233 brain and 233 cell line transcripts from African (NA18507) novel sequences could be aligned to GRCh37, revealing that some genes are absent from NCBI build 36, and part of them have been refined in GRCh37. In sum, we observed 4.12% (58.061 kb of expressed 211.025 kb regions could be aligned to GRCh37) Asian (YH) and 4.19% (49.293 kb of expressed 201.011 kb could be aligned to GRCh37) African (NA18507) novel sequences transcribed in brain, and 5.49% (78.581 kb out of 281.13 kb expressed regions could be aligned to GRCh37) Asian (YH) and 5.08% (56.485 kb out of 244.017 kb expressed regions could be aligned to GRCh37) African (NA18507) novel sequences transcribed in cell lines. Moreover, the ethnic group of brain donors (22 Caucasian and one unknown) indicates that many of these transcribed sequences are not specific to Asian (YH) and African (NA18507), but also present in Caucasian.

### Quantifying the expression of unalignable RefSeq genes

To gain insights into the expression profile of those human NCBI RefSeq genes that are unalignable to GRCh37, we first aligned the human NCBI RefSeq gene sequences onto GRCh37. We collected 176 RefSeq genes with identity < 90% or coverage < 95% to GRCh37, defining them as "unalignable RefSeq genes". The reason we chose 95% coverage as the threshold is that if a RefSeq gene sequence is 2 kb long in length, 5% deletion of its length is 100 bp. This small deletion can have a strong effect on its annotation on the reference genome and the number of reads mapping to this RefSeq gene. Next, we used Mortazavi's approach [25] to estimate the expression level of these unalignable RefSeq genes in brain and cell lines (see Methods). Using 0.1 RPKM as threshold, we observed 85 unalignable RefSeq genes expressed in brain and 93 unalignable RefSeq genes expressed in cell lines, with 74 unalignable RefSeq genes in common (Additional file 5 Figure S1). Among these unalignable expressed RefSeq genes, 60 from brain (70.59%) and 67 from cell lines (72.04%) are hypothetical genes with unknown functions, the rest are from different gene families including phospholipase inhibitor LOC646627, non-coding RNA NCRNA00107, neuregulin NRG1 (Additional files 6 and 7). For those unalignable expressed RefSeq genes curated in NCBI (with "NM-" and "NR-" prefix), they have diverse important functions. For example, the KCNIP4 gene, which is the regulatory subunit of Kv4/D (Shal)-type voltage-gated rapidly inactivating A-type potassium channels, is annotated in NCBI as partial on reference assembly. Another example is the FGF16 gene, which is a member of the fibroblast growth factor (FGF) family and is involved in a variety of biological processes, including embryonic development, cell growth, morphogenesis, tissue repair, tumor growth and invasion, but its annotation category in NCBI also belongs to the partial on reference assembly. Those unalignable expressed RefSeq genes could result from the misassembly of the human reference genome and their absence will restrict our understanding of the complete structure, the function of the human genome, and limit our interpretation of the related biology processes those genes are involved in.

To check whether those unalignable expressed RefSeq genes in brain and cell lines are conserved, we mapped them onto chimpanzee and macaque genomes, respectively. Using 90% identity and 90% coverage as threshold, 60 unalignable expressed RefSeq genes could be aligned to chimpanzee and 51 unalignable expressed RefSeq genes could be aligned to macaque for brain sample (45 are in common); while 64 unalignable expressed RefSeq genes could be aligned to chimpanzee and 62 unalignable expressed RefSeq genes could be aligned to macaque for cell lines (52 are in common). Among those 45 unalignable expressed brain RefSeq genes and 52 unalignable expressed cell line RefSeq genes that are aligned to both chimpanzee and macaque, 42 are in common. The results signify that those unalignable expressed RefSeq genes that could be both mapped to chimpanzee and macaque genomes are highly conserved across human, chimpanzee and macaque, indicating their potential significant function and biological importance. Among the unalignable expressed RefSeq genes between brain and cell lines, 75 have been annotated as predicted with unknown functions, we then mapped those 75 human RefSeq genes to the RefSeq genes of chimpanzee and macaque. Using Blast with a cut-off of E-value < 10^-5 ^and identity > 80%, we found 73 predicted human RefSeq genes matching with the RefSeq genes of chimpanzee and macaque, but most of those matched RefSeq genes of chimpanzee and macaque are also annotated as predicted and only two of them have related functional annotation (both of them are from chimpanzee, one is marked as the nuclear factor of kappa light polypeptide gene enhancer in B-cells inhibitor-like 1 (NFKBIL1) and another is autophagy related 13 homolog (ATG13)). The NFKBIL1 gene may be a negative regulator of NF-kappa-B activation. The ATG13 gene has been annotated as an autophagy factor required for autophagosome formation and it is conserved in human, chimpanzee, mouse, dog, cow, rat, chicken and zebrafish.

**Figure 2:**
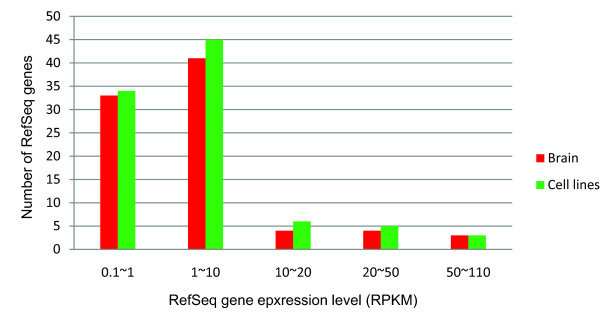
**The expression levels of those unalignable RefSeq genes in brain and cell lines**. The threshold is 0.1 RPKM (**R**eads **P**er **K**ilobase of the transcript per **M**illion mapped reads).

These results suggest that when based on only the incomplete human reference genome, it will certainly miss many expressed genes not presented in the reference genome. As we can see from Figure 2, the expression levels of some unalignable RefSeq genes are high, indicating that those unalignable RefSeq genes are active in brain or cell lines. Over two thirds of these unalignable expressed RefSeq genes in brain and cell lines are hypothetical genes and may have important unknown functions. Therefore, it would be essential to examine the expression profile of those genes and elucidate their biological functions through various experimental and/or computational approaches in the future.

### *De novo *assemblies of brain and cell line transcriptome

To examine whether any other novel gene expressed beyond the human reference genome, human RefSeq genes and EST sequences, we carried out *de novo *short-read assemblies using velvet [26] on both brain and cell line transcriptome sequencing data. Since the assembly errors are usually enriched in the short and low coverage assembly contigs, we removed the contigs with length < 60 bp and coverage < 5. Using this threshold, we obtained 254,769 and 204,625 transcript contigs from brain and cell lines, respectively (Additional file 5, Table S3). The N50 contig size is 284 bp for brain and 394 bp for cell lines, the max contig size is 3,873 bp for brain and 6,812 bp for cell lines.

We then aligned [20] brain and cell line contigs onto GRCh37, human NCBI RefSeq genes and EST sequences with 90% identity and 90% coverage as threshold. After removing the contigs that can be aligned to them, 16,225 brain and 11,638 cell line contigs were left unalignable (Table 1), we denoted them as "unmapped contigs", and used them to explore novel transcript contigs.

**Table 1:** Novel transcript contigs in brain and cell lines.

Items	Brain	Cell lines
Total number of transcript contigs	254769	204625
Number of contigs unaligned to GRCh37, RefSeq genes and EST	16225	11638
Number of unmapped contigs aligned to Human Fosmid sequences	41	25
Number of unmapped contigs aligned to HuRef genome	184	100
Number of unmapped contigs aligned to Celera genome	181	90
Number of unmapped conitgs aligned to YH novel sequences	103	42
Number of unmapped contigs aligned to NA18507 novel sequences	137	46
Number of unmapped contigs aligned to chimpanzee genome	119	56
Number of unmapped contigs aligned to macaque genome	64	36
Total number of aligned unmapped contigs	313	173
Total contig length (bp)	50324	29664
N50 contig size (bp)	195	194

*The brain and cell line transcript contigs were aligned to the human reference genome (GRCh37), RefSeq genes and EST sequences with 90% indentify and 90% coverage as threshold. The unalignable brain and cell line transcript contigs were then aligned to human Fosmid sequences, HuRef genome, Celera genome, Asian (YH) and African (NA18507) novel sequences, chimpanzee and macaque genomes with 90% identity and 100% coverage as the threshold.

### Identifying novel transcript sequences

To determine how many of those unmapped contigs were truly novel transcript contigs in brain and cell lines, we first aligned them to human Fosmid sequences, HuRef [27] genome, Celera genome, the Asian (YH) and African (NA18507) novel sequences. With the same criteria of > = 90% identity and 100% coverage, 294 brain (61 bp ~ 1,304 bp in length) and 158 cell line (61 bp ~ 821 bp) unmapped contigs could be aligned to these human sequences or genomes(Table 1). We further aligned these unmapped contigs of brain and cell lines to chimpanzee and macaque genomes. Using 90% identity and 100% coverage as threshold, we found that 119 brain (61 bp ~ 1,233 bp in length) and 56 cell line (61 bp ~ 684 bp) unmapped contigs can be aligned to chimpanzee genome; 64 brain (61 bp ~ 448 bp) and 36 cell line (68 bp ~ 594 bp) unmapped contigs can be aligned to macaque genome (Table 1). Among them, 36 brain and 22 cell line unmapped contigs are in common between chimpanzee and macaque, suggesting that those novel transcript contigs are conserved among human, chimpanzee and macaque. Besides those novel transcript contigs that can be aligned to human Fosmid sequences, HuRef genome, Celera genome, Asian (YH) and African (NA18507) novel sequences, 10 brain (70 bp ~ 239 bp) and 8 cell line (141 bp ~ 528 bp) unmapped contigs can only be aligned to the chimpanzee genome, and 11 brain (127 bp ~ 240 bp) and 9 cell line (96 bp ~ 528 bp) unmapped contigs can only be aligned to the macaque genome.

In all, we identified 313 brain (61 bp ~ 1,304 bp, 50,324 bp in total, N50 contig size is 195 bp) and 173 (61 bp ~ 821 bp, 29,664 bp in total, N50 contig size is 194 bp) cell line novel transcript contigs that are unalignable to GRCh37, RefSeq genes and EST sequences (Additional files 8 and 9). We then mapped those identified brain and cell line novel transcript contigs to the RefSeq genes of chimpanzee and macaque, using Blast with the cut-off of E-value < 10^-5 ^and identity > 80%, 55 brain and 52 cell line novel transcript contigs can find homologs from the RefSeq genes of chimpanzee and macaque. But all those matched RefSeq genes of chimpanzee and macaque have been annotated as predicted and still have no clear functions, suggesting that the related homologous genes of those novel transcript contigs are also not well annotated in chimpanzee and macaque.

Our identified novel transcribed sequences may be missed by the limitation of previous technologies or their low expression. We found that besides those novel transcript contigs that mapped to Asian (YH) and African (NA18507) novel sequences, many novel transcript contigs could still be aligned to either one of the HuRef genome, Celera genome, Fosmid sequence, chimpanzee and macaque genomes, or several of them. This indicates that some of those novel transcript contigs might be Caucasian specific transcribed sequences. We also found that some novel transcript contigs have high similarities (> 90% identity) among human, chimpanzee and macaque, implying that these novel transcript contigs are generated from conserved functional regions. In the end, the majority of brain and cell line unmapped contigs remain to be identified. Those unidentified contigs could result from the following aspects: the limited number of available human genomes; the stringent selection criteria in each step; sequence alterations in mRNAs from RNA editing [28]; variations in transcribed regions (e.g. SNPs, indels, structural variation); or the assembly errors in contigs due to the limitation of assembly algorithms [29] and etc.

### Locating the positions of novel transcript contigs

To determine the location of those novel transcript contigs on the human chromosomes, we used HuRef and Celera two human genomes as references to map them. Among those novel transcript contigs, 212 brain (61 bp ~ 1,304 bp in length) and 112 cell line (61 bp ~ 821 bp) contigs can be aligned to HuRef or Celera genomes. 147 brain and 83 cell line contigs can be only found an unique match location on HuRef or Celera chromosomes (Additional file 5, Table S4), yet some of the remaining contigs can be aligned to more than one position (they might be transcribed from the repetitive or homologous regions or belong to CNVs), and others can be mapped onto the human unplaced genomic contigs.

To further position those novel transcript contigs onto GRCh37, we extended 10 kb on both sides of those contigs with HuRef genome as the reference and then mapped the extended sequences onto GRCh37. Through alignment, we found that some of those novel transcript contigs could result from the small deletions of GRCh37, some other contigs could be transcribed from the large deletions (> 1 kb) of GRCh37 (Table 2). However, we still could not find the appropriate locations for the rest extended sequences on GRCh37, and these contigs could be generated from the huge deletions of GRCh37.

**Table 2:** The locations of seven conserved brain novel transcript contigs on GRCh37.

Contigs	Length (bp)	Chromosomes	Estimated deletions (kb)	Deletion start coordinates
NODE_319067	163	Chr3	5.2	90651858
NODE_373345	100	Chr9	2.4	18778464
NODE_445444	138	Chr14	1.6	82423894
NODE_463359	131	Chr8	27.3	58365531
NODE_469290	100	Chr5	14.7	2864231
NODE_518716	100	Chr18	20.4	12912086
NODE_559864	100	Chr12	2.2	2903843

*The "estimated deletions" were calculated by aligning the extended contigs (extended 10 kb on both sides of the identified novel contigs with HuRef genome as the reference) onto GRCh37 to find the distance between two broken sequences of each extended contigs.

### RT-PCR validation of conserved novel transcript contigs

**Figure 3:**
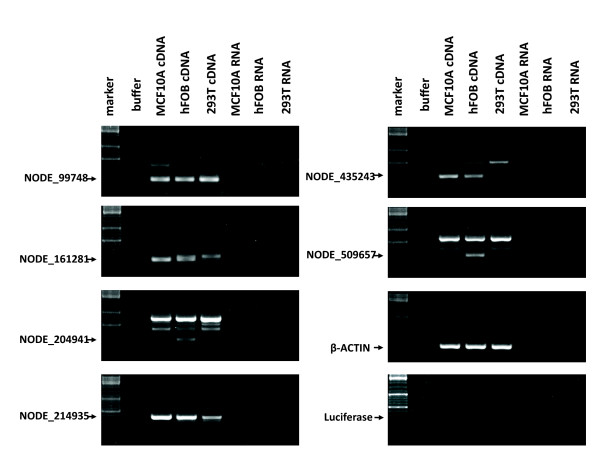
**RT-PCR validating of conserved novel transcript contigs**. Six conserved novel transcript contigs were validated expressed in three different types of human normal cells. Because gene expression usually exhibit temporal and spatial specificity, not all those novel transcript contigs were validated in every type of normal human cells. MCF10A: normal human breast cell; hFOB: human fetal osteoblast; 293T: human embryonic kidney cell; β-ACTIN: positive control; Luciferase: negative control; Marker: sm0331 DNA Ladder Mix.

In the brain and cell lines, 28 brain and 20 cell line novel transcript contigs can be aligned to HuRef, Celera, chimpanzee and macaque genomes, indicating that those contigs were transcribed from the conserved regions and might be functional. We then chose 10 of the conserved novel transcript contigs longer than 140 bp to validate their expression using RT-PCR, six of them were detected expressed in three different types of human normal cells (Figure 3). The experiment results further confirmed the reliability of our identification of those novel transcript contigs.

## Discussion

In this study, we detected a significant number of human genes and novel transcript contigs are still missing from the reference genome with human brain tissues and 10 mixed cell lines two transcriptome sequencing datasets. We verified that over 4% of Asian (YH) and 5% of African (NA18507) previously identified novel sequences that are absent from NCBI build 36 can be transcribed in brain and cell lines, and many of those transcribed regions might be not specific to Asian (YH) and African (NA18507), but also present in Caucasian. We estimated the expression levels of 176 human NCBI RefSeq genes that are unalignable to GRCh37, and found that about half of these unalignable RefSeq genes expressed in brain or cell lines, and some of them have been annotated with important functions. The majority of those unalignable expressed RefSeq genes are previously predicted by automated computational analysis with unknown functions, suggesting they have not been well studied yet. Further analysis shows that over 50 of those unalignable expressed RefSeq genes are highly conserved across human, chimpanzee and macaque, indicating their importance in human biological processes. Last, we carried out *de novo *transcriptome assemblies on both brain and cell line transcriptome, and identified hundreds of brain and cell line novel transcript contigs that cannot be aligned to NCBI build 37, RefSeq genes and EST sequences, indicating that there are still missing genes expressed beyond them. Some of those novel transcript contigs are conserved among human, chimpanzee and macaque, and they might be transcribed from the functional genes that absent from the human reference genome. By locating those contigs onto the human genome, we found some large deletions of human reference genome. Using RT-PCR, we validated six of those conserved novel transcript contigs expressed in three different types of normal human cells.

Our findings show that about a hundred RefSeq genes that are unalignable to GRCh37 are expressed and a significant number of our identified novel transcript contigs could be positioned to GRCh37, suggesting that those RefSeq genes and novel transcript contigs were from the gaps or misassembled regions of GRCh37. Some of our identified novel transcript contigs could be found more than one match locations on HuRef or Celera genomes, indicating that those contigs might be caused by CNVs or were transcribed from the repetitive or homologous sequences. In addition, there are still a portion of identified novel transcript contigs that could not be located to GRCh37 or HuRef or Celera genomes, implying that some of them might be specific to Caucasian and belong to the population or individual specific transcript sequences. Accordingly, due to the limitations of technologies and computational approaches, many reasons could lead to specific sequences missing from the human reference genome. These include: (i) the collection of the sequencing samples might be incomplete or even contaminated; (ii) the different sequencing technologies have certain bias in genome sequencing, and some genomic regions like AT-rich and GC-rich regions are difficult to sequence [30,31]; (iii) the genomic sequences, especially mammalian genomes are very complex and might contain large amounts of repetitive and homologous sequences; (iv) the assembly algorithms also have limitations for whole genome assembling and hardly assemble the sequencing reads into complete sequences [32]; (v) individual specific sequences could also contribute to the unalignable phenomena to the human reference genome and etc.

The sequencing technologies are undergoing fast development, it is expected that sequencing accuracy, read length and sequencing depths will be greatly improved [33,34]. In addition, many powerful bioinformatics algorithms are expected to be developed. These new developments will enable us to explore the structure of human genome more comprehensively and accurately, and realize personalized medicine in the near future.

## Conclusions

Our results indicate that there are still a significant amount of human genes not incorporated into the human reference genome, and their absence would result in the incomplete recognition of human genomics or transcriptomics. Our findings also show the importance of *de novo *assembly of transcriptome, which could help us to further explore the missing sequences beyond the reference genome. In addition, our study indicates that comparative analysis of human reference genome with other assembled human genomes provides an alternative approach to refine the human reference genome. To thoroughly explore and comprehensively understand the human genome, it is crucial to continue to refine the human reference genome, and to identify and characterize the genes that are not present in the reference genome.

## Methods

### Public data usage

We obtained the human genome reference assembly GRCh37 (NCBI build 37.1), human Fosmid sequences, HuRef assembly, Celera assembly, human NCBI RefSeq gene sequences, human EST sequences, the RefSeq gene sequences and genomes of chimpanzee (NCBI build 2) and macaque (NCBI build 1) from NCBI http://www.ncbi.nlm.nih.gov/guide/. We also downloaded the Asian (YH) and African (NA18507) novel sequences [13] that are absent NCBI build 36 from http://www.nature.com/nbt/journal/v28/n1/abs/nbt.1596.html.

### RNA-Seq data production

We used two transcriptome sequencing datasets from two reference RNA samples established by the MicroArray Quality Control (MAQC) project [18] with Illumina Genome Analyzer II. The two reference RNA samples consist of the Universal Human Reference RNA (UHRR or UHR, Catalog #740000) from 10 human cell lines of various origins: Blymphocyte, brain, breast, cervix, liposarcoma, liver, macrophage, skin, testis and T-lymphocyte [19] and the Human Brain Reference RNA (HBRR or HBR, Catalog #6050) from several regions of the brain of 23 adult donors (22 of these donors were Caucasian, one is unknown). Equal quantities of DNAase-treated total RNA from each cell line were pooled to generate the UHRR.

The cell line and brain datasets consist of ~70.1 million and ~58.6 million of stranded sequencing reads, respectively, with read-lengths of 100 bp. Those two datasets from this study have been submitted to the NCBI Gene Expression Omnibus http://www.ncbi.nlm.nih.gov/geo under accession number GSE30222.

### Detection of the transcribed regions in Asian and African novel sequences

We aligned the Asian (YH) and African (NA18507) novel sequences to GRCh37 using BLAT [20] with -fastmap option enabled. If a sequence can map to GRCh37 with > = 90% identity and > = 98% coverage, we deemed that it could be aligned to human reference genome. To detect the transcribed regions in Asian (YH) and African (NA18507) novel sequences, we first predicted the potential genes in these sequences using Augustus [21] program (version 2.4) with default parameters. Next, we mapped brain and cell line reads onto Asian (YH) and African (NA18507) novel sequences with Bowtie [22] program (version 0.12.3) and used TopHat [23] program (version 1.0.13) to detect the splice junctions between exons with default parameters. Next, we carried out transcript assembly and quantification through Cufflinks [24] program (version 0.8.3) with default parameters, and used Asian (YH) and African (NA18507) novel sequences as the reference. Any transcript with a confidence interval with FPKM (**F**ragments **P**er **K**ilobase of exon model per **M**illion mapped fragments) = 0 as the lower bound is highly suspicious and considered as unexpressed.

### Quantification of the expression of unalignable RefSeq genes

We used the Mortazavi's approach [25] to estimate the expression level of the unalignable human NCBI RefSeq genes that could not be aligned to GRCh37 with 90% identity and 95% coverage as threshold in RPKM (**R**eads **P**er **K**ilobase of the transcript per **M**illion mapped reads). The RPKM formula is as follows:

$$RPKM=10^{9}\times \frac{C}{N*L}$$

Where C is the number of mappable reads mapped onto the gene's exons, N is the total number of mappable reads in the experiment, and L is the total length of the exons in base pairs. In process, we removed the poly-A tails of the unalignable RefSeq gene sequences if the number of base "A" of the sequence is over ten. To avoid the influence of the partial sequences of some unalignable RefSeq genes on GRCh37, we first mapped the brain and cell line reads onto GRCh37 and the unalignable RefSeq genes to obtain the total number of mappable reads, and then we mapped brain and cell line reads onto the unalignable RefSeq genes to obtain the number of mappable reads that fell onto the gene's exons. We used Bowtie and TopHat with default parameters to complete these processes, and found the total number of mappable reads and the number of mappable reads that fell onto the unalignable gene's exons.

Those unalignable RefSeq genes with expression level lower than 0.1 RPKM were recognized as unexpressed. We further mapped those expressed unalignable RefSeq genes in brain and cell lines to chimpanzee and macaque genomes using BLAT with -fine option enabled to investigate the conservation of those genes, respectively.

### *De novo *short-read assembly

The assemblies of human brain and cell line transcriptome were performed by Velvet [26] software (version 0.7.60) with -strand-specific option enabled. We set the hash length k = 31 and coverage cut-off value as 5. The relation between k-mer coverage *C_k _*and standard (nucleotide-wise) coverage *C *is *C_k _*= C*(*L*-*k*-1)/*L*. Where k is the hash length, *C_k _*is how many times a k-mer has been detected among the reads and L is the read length. Very short and very low coverage contigs are likely errors, the contigs shorter than 2k-1 (60 bp) and coverage < 5 were removed.

### Identification of novel transcript contigs

We used BLAT program to align brain and cell line contigs. The contigs were first aligned to GRCh37, human NCBI RefSeq genes and human EST sequences to remove the alignable contigs with the threshold of 90% identity and 90% coverage. The unalignable contigs from brain and cell lines were then mapped to human Fosmid sequences, HuRef genome, Celera genome, the Asian (YH) and African (NA18507) novel sequences, chimpanzee genome and macaque genome. Only the contigs with > = 90% identity and 100% coverage were regarded to be expressed in the brain or cell lines and were considered as the novel transcript contigs.

### RNA isolation, reverse transcription and PCR

Total RNA was extracted using Trizol Reagent (invitrogen), and then each RNA sample was reverse transcribed using PrimeScript RT reagent Kit (Takara). All of these operations were carried out according to the manufacture's instruction. Polymerase chain reaction to amplify cDNA fragments was performed 35 cycles at 94°C/30s, 48°C/30s, and 72°C/30s for per cycle. Primers for PCR could be found in Additional file 5.

## Competing interests

The authors declare that they have no competing interests.

## Authors' contributions

TLS, LMS and GC designed the study. GC and TLS wrote the manuscript. GC, RYL and PZH performed the statistical and computational analyses. JYQ did the RT-PCR experiments, JL and MYL designed and supervised the experiments. All authors read and approved the final manuscript.

## Supplementary Material

Additional file 1 **Predicted transcripts in Asian (YH) novel sequences using brain RNA-Seq reads**. Click here for file

Additional file 2 **Predicted transcripts in Asian (YH) novel sequences using cell line RNA-Seq reads**. Click here for file

Additional file 3 **Predicted transcripts in African (NA18507) novel sequences using brain RNA-Seq reads**. Click here for file

Additional file 4 **Predicted transcripts in African (NA18507) novel sequences using cell line RNA-Seq reads**. Click here for file

Additional file 5 **Supplementary information includes supplementary figures, tables, the primers of RT-PCR and the instructions of supplementary files**. Click here for file

Additional file 6 **(Supplementary Table S1) The expression levels of unalignable human NCBI RefSeq genes in brain**. Click here for file

Additional file 7 **(Supplementary Table S2) The expression levels of unalignable human NCBI RefSeq genes in cell lines**. Click here for file

Additional file 8 **Identified novel transcript contigs in human brain tissues**. Click here for file

Additional file 9 **Identified novel transcript contigs in 10 mixed cell lines**. Click here for file
