# Supplementary material for: Revealing the missing expressed genes beyond the human reference genome by RNA-Seq
Source: BMC Genomics. 2011 Dec 2;12:590. doi: 10.1186/1471-2164-12-590 (PMC3288009; doi:10.1186/1471-2164-12-590)
Supplement: Additional file 5 — Supplementary information includes supplementary figures, tables, the primers of RT-PCR and the instructions of supplementary files. [file 1471-2164-12-590-S5.DOC]

**Supplementary information**

**Experiments**

We validated 6 cell line conserved novel transcript contigs expressed in three different types of human normal cells (human normal breast cell, human fetal osteoblast and human embryonic kidney cell) by PR-PCR.

The names and primers of those validated conserved novel transcript contigs are as follows:

1. NODE_99748_length_114_cov_7.622807 Chr10

Primer:

F 5' AAGGGCTGAGATCCATGTTT 3'

R 5' GCCTAACGACACATTGCTCA 3'

1. NODE_161281_length_229_cov_12.580786 Chr17

Primer:

F 5' GTTGGGACATGGTAAGAG 3'

R 5' GTGCCTTCACCCTACTTTAT 3'

1. NODE_204941_length_564_cov_24.409575 Chr7

Primer:

F 5' CCAAGAAGTGTTTGAGGG 3'

R 5' CATTGAAGGGTAGTGAAGAG 3'

1. NODE_214935_length_425_cov_20.249413 Chr17

Primer:

F 5' CTCACATCACGGACAAAG 3'

R 5' ATGTTTACCCTCCCCTAC 3'

1. NODE_435243_length_221_cov_5.515837 Chr17

Primer:

F 5' GGATTCGTGGGAGACTAT 3'

R 5' TGTGGGGACTCTTTTCAGG 3'

1. NODE_509657_length_480_cov_5.272917 Chr7

Primer:

F 5' GCATTTCCTGCTTGATACTG 3'

R 5' GTTTGAGACATGGACTTTGG 3'

Luciferase Primer:

F 5' ACAGTATGGGCATTTCGC 3'

R 5' CCTTTAGGCAGACCAGTAGA 3‘

β-ACTIN Primer:

F 5' GTACGCCAACACAGTGCTG 3‘

R 5' CGTCATACTCCTGCTTGCTG 3‘

**Supplementary figures**

**Figure S1 Unalignable expressed RefSeq genes in brain and cell lines (threshold: 0.1 RPKM)**. **A** The number of expressed unalignable RefSeq genes in brain and cell lines. Unalignable RefSeq genes are those RefSeq genes that with <90% identity or <95% coverage to the human reference genome (GRCh37); **B, C** The composition of expressed unalignable RefSeq genes in brain and UHR. Predicted RefSeq genes represent those RefSeq genes that have not been reviewed with unknown function. Validated RefSeq genes represent those RefSeq genes that have undergone validation or preliminary review. Reviewed RefSeq genes represent those RefSeq genes that have been curated by NCBI staff.

**Supplementary tables**

**Table S1:** **The expression levels of unalignable human NCBI RefSeq genes in brain.**

(Attached Excel file)

**Table S2: The expression levels of unalignable human NCBI RefSeq genes in cell lines.**

(Attached Excel file)

**Table S3: Statistics of brain and cell lines transcriptome assemblies.**

| **Samples** | **Read length (bp)** | **Number of reads** | **Number of contigs** | **N50 contig size (bp)** | **Max contig size (bp)** |
| --- | --- | --- | --- | --- | --- |
| Brain | 100 | 58,578,322 | 254,769 | 284 | 3,873 |
| Cell lines | 100 | 70,105,327 | 204,625 | 394 | 6,812 |

**Table S4:** **The location of brain and cell line novel transcript contigs.**

| **Chromosome** | **Number of brain novel transcript contigs** | **Number of cell line novel transcript contigs** |
| --- | --- | --- |
| Chr1 | 6 | 9 |
| Chr2 | 4 | 1 |
| Chr3 | 8 | 1 |
| Chr4 | 9 | 1 |
| Chr5 | 5 | 1 |
| Chr6 | 7 | 1 |
| Chr7 | 19 | 13 |
| Chr8 | 8 | 2 |
| Chr9 | 0 | 0 |
| Chr10 | 6 | 5 |
| Chr11 | 10 | 4 |
| Chr12 | 10 | 3 |
| Chr13 | 4 | 4 |
| Chr14 | 4 | 3 |
| Chr15 | 4 | 3 |
| Chr16 | 6 | 5 |
| Chr17 | 13 | 12 |
| Chr18 | 2 | 4 |
| Chr19 | 22 | 11 |
| Chr20 | 0 | 0 |
| Chr21 | 0 | 0 |
| Chr22 | 0 | 0 |
| ChrX | 0 | 0 |
| ChrY | 0 | 0 |

**Supplementary Files**

**File S1:** Predicted transcripts in Asian (YH) novel sequences using brain RNA-Seq reads.

**File S2:** Predicted transcripts in Asian (YH) novel sequences using cell line RNA-Seq reads.

**File S3:** Predicted transcripts in African (NA18507) novel sequences using brain RNA-Seq reads.

**File S4:** Predicted transcripts in African (NA18507) novel sequences using cell line RNA-Seq reads.

**Supplementary Datasets**

**Dataset S1:** (Additional file 8) Identified novel transcript contigs in human brain tissues.

Novel brain transcript contigs that are unalignabe to NCBI build 37, RefSeq genes and EST sequences, but could be aligned to one of these sequences: human Fosmid sequences, HuRef genome, Celera genome, the Asian (YH) and African (NA18507) novel sequences, chimpanzee genome and macaque genome with 90% identity and 100% coverage as threshold.

**Dataset S2** (Additional file 9) Identified novel transcript contigs in 10 mixed cell lines.

Novel cell line transcript contigs that are unalignabe to NCBI build 37, RefSeq genes and EST sequences, but could be aligned to one of these sequences: human Fosmid sequences, HuRef genome, Celera genome, the Asian (YH) and African (NA18507) novel sequences, chimpanzee genome and macaque genome with 90% identity and 100% coverage as threshold.
